# Multi-Function of a New Bioactive Secondary Metabolite Derived from Endophytic Fungus *Colletotrichum acutatum* of *Angelica sinensis*

**DOI:** 10.4014/jmb.2206.06010

**Published:** 2022-12-27

**Authors:** Ramy S. Yehia

**Affiliations:** 1 Department of Biological Sciences, College of Science, King Faisal University, Al-Ahsa 31982, Saudi Arabia; 2 Department of Botany and Microbiology, Faculty of Science, Cairo University, Giza 12613, Egypt

**Keywords:** Endophytic fungi, *Angelica sinensis*, *Colletotrichum acutatum*, biological activities

## Abstract

In the current study we assessed a new crystallized compound, 5-(1-hydroxybutyl)-4-methoxy-3-methyl-2*H*-pyran-2-one (C-HMMP), from the endophytic fungus *Colletotrichum acutatum* residing in the medicinal plant *Angelica sinensis* for its in vitro antimicrobial, antibiofilm, antioxidant, antimalarial, and anti-proliferative properties. The promising compound was identified as C-HMMP through antimicrobial-guided fraction. The structure of C-HMMP was unambiguously confirmed by 2D NMR and HIRS spectroscopic analysis. Antimicrobial property testing of C-HMMP showed it to be effective against a variety of pathogenic bacteria and fungi with MICs ranging from 3.9 to 31.25 μg/ml. The compound displayed excellent antibiofilm activity against *C. albicans*, *S. aureus*, and *K. pneumonia*. Furthermore, the antimalarial and radical scavenging activities of C-HMMP were clearly dosede-pendent, with IC50 values of 0.15 and 131.2 μg/ml. The anti-proliferative activity of C-HMMP against the HepG-2, HeLa, and MCF-7 cell lines in vitro was investigated by MTT assay, revealing notable anti-proliferative activity with IC50 values of 114.1, 90, and 133.6 μg/ml, respectively. Moreover, C-HMMP successfully targets topoisomerase I and demonstrated beneficial anti-mutagenicity in the Ames test against the reactive carcinogenic mutagen, 2-aminofluorene (2-AF). Finally, the compound inhibited the activity of α-glucosidase and α-amylase with IC50 values of 144.7 and 118.6 μg/ml, respectively. To the best of our knowledge, the identified compound C-HMMP was obtained for the first time from *C. acutatum* of *A. sinensis*, and this study demonstrated that C-HMMP has relevant biological significance and could provide better therapeutic targets against disease.

## Introduction

The importance of natural products in drug discovery and development has been widely reported [1]. Drugs used to treat cancer, lipid disorders, infectious diseases, and hypertension [2] are examples of how important natural products are as sources of novel therapeutic agents. However, choosing the natural source to be evaluated is a vital factor in any successful natural product-based drug development program. Remember that novel chemical variety is frequently linked to untapped and/or untapped sources of biological diversity. In various medicinal and agricultural disciplines, endophytic fungi have been shown to produce a wide number of different structural and economically active secondary metabolites [3]. Functionally, endophytic fungi are identified by their presence in plant tissue without manifesting any harm [4], and their metabolic interactions with the environment expand the potential of bioactive product generation [5, 6]. New drugs for the efficient treatment of diseases in animals, humans, and plants can result from research into the metabolites produced by endophytic strains [6, 7]. Endophytes have the ability to produce more natural products, as seen by the growing number of novel compounds being found in them. These natural products have yet to be fully utilized for their prospective applications. Many bioactive compounds have key features that, when isolated and characterized, may also have potential use in medicine, industry, and agriculture [8]. These properties include antiviral, antioxidant, antimicrobial, anticancer, cytotoxic, immunomodulatory, antiparasitic, and insecticidal properties. Because of their extensive history of co-evolution and genetic recombination, the majority of endophytic fungi have metabolic abilities comparable to the related host [9]. Furthermore, the fact that these endophytes are symbiotic partners with their host plants suggests that their bioactive substances are less harmful to cells and do not destroy the system of eukaryotic host [10]. Only 6-7% of the estimated 1.5 million fungal species, including endophytes, have been described thus far; the remainder are currently awaiting inclusion in the known microbial world. Moreover, it is thought that 51% of the bioactive compounds identified from these endophytic fungi were previously unknown [11]. Therefore, this work is focused on isolating and characterizing new bioactive molecules from the endophytic fungi harboring in *Angelica sinensis*, as well as assessing their potential anticancer, antioxidant, antibiofilm, antimutagenic, antimalarial, antidiabetic, and antimicrobial activities. Furthermore, our goal in this research is to establish endophytic fungi as viable natural repositories for the identification of novel drugs with potential applications in the food, pharmaceutical, and agricultural sectors.

## Materials and Methods

### Collection of Samples

*Angelica sinensis* (family: Apiaceae) was procured in April 2021 from the Al-Ahsa region of Saudi Arabia (25°51¢7571, 49°77¢8137) in order to isolate endophytic fungi. Within 12 h after collection, healthy plant samples were firmly packed in polyethylene bags and processed for isolation of fungi.

### Isolation and Identification of Endophytes from Plant Samples

The surfaces of the leaf samples were sterilized by dipping them in 70% ethanol for 10 min, 2.5% sodium hypochlorite in 0.1% Tween 20 Detergent (TBST, Merck, Germany) for 15 min, and then thoroughly washed five times with double-distilled deionized sterile water. This process removed soil particles and microorganisms adhering to the surfaces of the leaves. After properly sterilizing the leaves, the sections were cut into 2 cm lengths and aseptically placed in potato dextrose agar (PDA; Laboratories Conda SA, Spain) plates that also included streptomycin (60 μg/ml) to inhibit the growth of bacteria. For 5-8 days, the plates were incubated at 28°C and growth was checked every day. To create a pure culture for identification and enumeration, the hyphal tips emerging from the sterile tissues were sub-cultured onto a new cultural media.

Following sporulation on cultivation media, the recovered pure fungal isolates were initially identified using their morphological traits in accordance with conventional identification guidelines [12-14]. The percentage of colonization frequency (CF%)‒shown below and defined as the proportion of leaf fragments producing an endophytic fungi in culture [15]‒was used to analyze the endophytic fungal isolates from plant tissue segments.

$$\text{CF =}\frac{\text{number of segments colonized by fungi}}{\text{total number of segments observed}}\times 100$$

Moreover, the fungal dominance percentage (DF%) was calculated as:

$$\text{DF =}\frac{\text{percentage colony frequency}}{\text{the sum of the percentage of colony frequency of all fungi}}\text{×100}$$

For genotypic identification, the entire genomic DNA of the most promising endophytic fungi was extracted using a DNA extraction kit (Qiagen, Hilden, Germany) directly from mycelium that was actively growing in potato dextrose broth (PDB; HiMedia, India). Five loci, including the partial actin (ACT) gene, partial glyceraldehyde-3-phosphate dehydrogenase (GAPDH) gene, partial chitin synthase (CHS) gene, large subunit ribosomal RNA (28S rRNA), and internal transcribed spacers (ITS), were amplified using the respective primer sets, ITS1 TCCGTAGGTGAACCTGCGG/ITS4: TCCTCCGCTTGATATGC [16], 28S rRNA: ACCCGCTGAACTTAAGC/TCCTGAGGGAAACTTCG [17], ACT: ACT-512F: ATGTGCAAGGCCGGTTTCGC, ACT-738R: TACGAG TCCTTCTGGCCCAT, GAPDH: GD-F1: GCCGTCAACGACCCCTTCATTGA, GD-R1: GGGTGGAGTCGT ACTTGAGCATGT, respectively. The BigDye Deoxy Terminator Cycle-Sequencing Kit (Applied Biosystems, Germany) was used to sequence all products using an automated DNA sequencer (ABI PRISM 3700, Germany). To identify the closest sequences for taxonomic framework, the sequences generated during the current investigation were blasted against the NCBI database (http://www.ncbi.nlm.nih.gov.blast). The GenBank database was filled with the consensus sequences. Each locus' GenBank accession numbers were collected, and MEGA v.7 software was used for phylogenetic analysis.

### Extraction of Secondary Metabolites

Pure endophytic fungal isolate with the highest percentage of colonization frequency was selected for seed production of secondary metabolites. In 250-ml Erlenmeyer flasks containing 100 ml potato dextrose broth (PDB) each, two to three plugs of agar medium (0.5 × 0.5 cm) were inoculated and incubated at 28°C for 7 days while being shaken at 150 rpm. Twenty Erlenmeyer flasks (1 L, pH 5.5) were used for the scale-up batch level fermentation. To each flask containing 500 ml of broth, 10% (w/v) of prepared mycelial inoculum was transferred and incubated at 28°C in the dark for two weeks. After batch fermentation was completed, the mycelia-free broth was collected by filtering, and metabolites were extracted by pouring 150 ml of ethyl acetate (EtOAc) into each flask and vigorously mixing. The extract of EtOAc was then condensed in a rotary evaporator (IKA, Germany), followed by freeze-drying. The total EtOAc extract was collected for further analysis.

### Separation of Bioactive Compound

By using column chromatography, 450 mg of fungal EtOAc extract of *C. acutatum* (from *A. sinensis*) was sequentially fractionated. The column was loaded with silica gel (30 g, 60–120 mesh size, Sigma-Aldrich, Japan), and endophytic fungal extract combined with 2.0 g silica powder. Hexane was used to elute the column first, followed by an increasing polarity by combination of hexane and ethyl acetate. Thin-layer chromatography (TLC) was used to verify the fractions obtained from column chromatography. Aluminum sheets coated with silica gel from Merck (Darmstadt, Germany), was used, and the spots were inspected under 254 and 366 nm UV light. The mobile phase was a mixture of chloroform, methanol, and ethyl acetate (9:3:5). The retention factor of the analyte provided an indication of its mobility (Rf). The crude was divided into four bands for this preliminary screening, and the fractions were then concentrated in a rotary evaporator, weighed, and tested for antimicrobial activity against different microbes.

### Antimicrobial Activity-Guided Fraction

As directed by the Clinical and Laboratory Standards Institute (CLSI) for bacterial protocol [20] and fungal protocol [21], the fractionated metabolites were tested against bacterial and fungal pathogens obtained from the Department of Plant Pathology, Faculty of Agriculture, Cairo University using disk diffusion assay. The crude crystallized fractions (R1, R2, R3, and R4) were diluted in 1% dimethyl sulphoxide (DMSO), and the sterilized disc (Whatman no. 5, 5-mm diameter, Sigma, USA) was loaded with the solution of 10 μl (1 mg/10 μl) to get promising secondary metabolites. The following gram-negative and gram-positive bacteria were tested for antibacterial activity: *Pseudomonas syringae*, *Xanthomonas oryzae*, *Aeromonas hydrophila*, and *Staphylococcus aureus*. On the surface of solidified Mueller-Hinton agar plates (HiMedia, India), the test bacterial cultures were created using a cotton swab. The Muller-Hinton agar medium was already inoculated with test bacteria in plates when sterile paper discs holding 10 μl of TLC fractions (1 mg/10 μl) were separately placed on the surface. As a negative and positive control, paper discs impregnated with 10 μl DMSO and streptomycin (50 μg per disc) were utilized. By evaluating the zone of inhibition, the plates were examined for antibacterial activity after being incubated for 24 h at 35 ± 2°C. On Sabouraud agar plates (g/l); dextrose, 40; peptone, 10; agar, 15 (HiMedia, India), the fractions were also tested for their ability to inhibit the growth of fungi, including *Aspergillus flavus*, *Fusarium solani*, *Candida albicans*, and *Trichophyton rubrum*. The plates were examined for antifungal activity after being incubated at 25 ± 2°C for 3-5 days. The zone of inhibition diameters were recorded for each experiment in triplicate and expressed in millimeters.

### Determination of Minimum Inhibitory Concentration

For selected bioactive fractions, the MIC values of resulting fractions against the aforementioned bacteria and fungi were assessed using broth microdilution procedures in a 96-well microplate [22] with minor modifications. In brief, 100 μl of two-fold diluted fractions with final concentrations ranging from 0.12 to 1,000 μg were added to 100 μl of 0.5 Macfarland bacterial cultures as an inoculum. MIC values for bacteria and fungi were determined after 24 h at 37°C and 96 h at 28°C, respectively. The MIC is defined as the lowest concentration at which growth fails to occur.

### Elucidation of Bioactive Compound Structure

Nuclear magnetic resonance (NMR) and high-resolution mass spectrometry (HRMS) investigations were used to identify the structural composition. Using the non-deuterated residual solvent signal as a reference, 1D-NMR (^1^H, 500 MHz; ^13^C, 125 MHz) and 2D-NMR (edited-HSQC, HMBC, NOESY, and COSY) studies were carried out using a Varian DRX-500 spectrometer. Parts per million (ppm) are used to express the chemical shifts. Using MeOH or MeOH/H2O as the eluent, mass spectra were obtained using a Bruker ultrOTOF-Q-ESI-TOF mass spectrometer (cone voltage: 25 V, Germany).

### Antioxidant Capacity Assay

With a few minor adjustments, the 2,2-diphenyl-1-picrylhydrazyl (DPPH)-free radical scavenging experiment was carried out in accordance with Zhao *et al*. [23]. The tested compound was diluted in ethyl acetate (100 μl) at different concentration of 10, 25, 50, 100, 150, 200, and 250 μg/ml before being added to DPPH (1 M, 100 μl, Sigma-Aldrich, USA). The negative control was pure ethyl acetate. A positive control, ascorbic acid (vitamin C) was employed. After providing the reaction mixture a good shake, it was left to rest at room temperature in the dark for 30 min. Each experiment was carried out three times. Using a UV-Vis spectrophotometer, the absorbance was checked at 517 nm after 30 min. The radical scavenging activity was calculated using the following formula:

$$\text{DPPH radical scavenging activity (\%) =}\frac{\text{As -Ab}}{\text{A0}}\times 100,$$

where As represents the absorbance of test samples, Ab represents the bank's absorbance (without DPPH) and A0 is the control's absorbance (without sample). The sample concentration needed to scavenge 50% of DPPH (IC50, 50% inhibitory concentration) was calculated.

### Antimutagenic Assay

The Maron and Ames [24] technique of utilizing the plate incorporation assay, with minor modifications, was used to determine the antimutagenic activity of an isolated compound. The Institute Pasteur in France donated the *Salmonella typhimurium* strain TA98. The investigated compound was prepared in various doses (0.05–1 mg) in 30% DMSO. To create a positive control, minimal agar plates were coated with top agar and then poured with 0.1 ml of the overnight-grown culture and 0.1 ml of the 2-aminofluorene (2 AF) mutagen. In the negative control, 5 ml of top agar was mixed with 0.1 ml of the fungal extract and 0.1 ml of the overnight-developed culture before being put onto minimal agar plates. To assess the toxicity/mutagenicity levels of the isolated compound, 0.1 ml of the overnight-developed culture and 0.1 ml of the tested compound were utilized.

Further, 0.5 ml of S9 mix (sterile distilled water 16.75 ml, 0.2 M sodium phosphate buffer (pH 7.4), 0.1 M NADP, 1 M G-6-P, MgCl_2_-KCl, Rat liver S9 (phenobarbitone-induced), 0.1 ml of 2-AF, and 0.1 ml of the isolated compound were added into top agar and poured onto the plates to calculate the percentage of inhibition. Colonies were enumerated after 48 h of incubation at 37°C with the total mixture. Every test was run in triplicate. The following formula was applied to compute the percentage of inhibition:

$$\text{\% of inhibition =}\frac{\text{A-B}}{\text{A-C}}\times 100,$$

where C represents the number of spontaneous colonies and A, B, and D represent the number of colonies in the positive control, negative control, and test sample, respectively.

### Antimalarial Assay

The isolated compound's antimalarial activity was assessed using a slightly modified version of the Budimulya *et al*. [25] technique. Briefly, 25 μl of the isolated compound was diluted in DMSO (10 μg/ml) and then stored at –20°C until usage. *Plasmodium chabaudi* (200 μl, malarial parasite) was provided by the Department of Zoology, King Saud University, Saudi Arabia, which was propagated in a 24-well culture plate with different concentrations (0.001, 0.01, 0.1, 1.0 and 10 μg/ml) and incubated at 37°C for 48 h. An IC50 value, which is the concentration of the tested compound that inhibits growth of parasite by 50% compared to the untreated control, was recorded. The standard antimalarial drug used in this approach is dihydroartemisinin, which has an IC50 of 0.0014 μg/ml.

The inhibition percentage was calculated as follows:

$$\text{(\% inhibition) =100\% -}\left[\frac{\text{Xu}}{\text{Xk}}\text{×100\%}\right]\text{,}$$

where Xu indicates the percentage of growth of each isolate (concentration), while Xk indicates the percentage of growth of negative control.

### Antidiabetic Activity

**α-Amylase Inhibition Assay**

The isolated compound from *C. acutatum* was evaluated for α-amylase inhibitory activity in accordance with Worthington [26], with minor modifications. Different concentrations of the test compound or positive control (acarbose) (10, 25, 50, 100, 150, 200, and 250 μg/ml) were mixed with 500 μl of a solution containing 1.0 U/ml of α–amylase in 0.02 M sodium phosphate buffer (pH 6.9 adjusted with 0.006 M NaCl) and incubated at 25°C for 10 min. The sodium phosphate buffer was then treated with 500 μl of 1% starch solution. For another 10 min, the fusion was incubated at 25°C. One milliliter of 3.5-dinitro salicylic acid (DNS) color reagent was applied to stop the reaction. The test tubes were cooled at 37°C after 5 min of incubation in a hot water bath.

After diluting the mixture with 10 ml of distilled water, the absorbance at 540 nm was measured using a UV-Vis spectrophotometer (Biophotometer Plus, Japan). The following formula was employed to calculate α-amylase inhibition:

$$\text{Inhibition(\%)=}\frac{\text{A(control)-A(sample)}}{\text{A control}}\times 100,$$

where, Acontrol denotes the absorbance of the control (devoid of the investigated compound), while Asample denotes the presence of the tested compound.

### α-Glucosidase Inhibition Assay

Further, with minor modifications from Zhang *et al*. [27], the isolated compound was tested for its ability to inhibit α-glucosidase. It was diluted in 100 μl of 0.1 M potassium phosphate buffer (pH 6.9) containing α-glucosidase (1.0 U/ml). On a 96-well plate, this mixture was incubated for 10 min at 25°C. To measure the absorbance at 406 nm, a UV-Visible spectrometer (Biophotometer Plus, Japan) was employed. Subsequently, 50 μl of 5 mM p-nitrophenyl–α– d-glucopyranoside (pNPG) in 0.1 M potassium phosphate buffer (pH 6.9) were added to each well. After incubating the sample at 25°C for 5 min, the absorbance was once more measured. The following formula was used to calculate the inhibitory activity of α-glucosidase, which was expressed as a percentage of inhibition:

$$\text{Inhibition(\%)=}\frac{\text{A(control)-A(sample)}}{\text{A (control)}}\times 100,$$

where, Acontrol represents acarbosés absorbance, while Asample represents the absorbance of tested compound. IC50; the concentration of tested compound that inhibited enzyme activity by 50% was estimated.

### Antibiofilm Activity

Three distinct microbes (*Candida albicans*, *Staphylococcus aureus*, and *Klebsiella pneumonia*) were obtained from the Botany and Microbiology Department at King Saud University in Saudi Arabia and employed in the study to determine the antibiofilm capabilities of a purified compound. In nutrient broth, bacteria were cultivated at 37°C. At 25°C, yeast malt broth medium was used to activate fungal strains. The 2,3-Bis-(2-methoxy-4-nitro-5-sulfophenyl]-2H-tetrazolium-5-carboxyanilide salt (XTT) assay, disruption of the preformed biofilm, and inhibition of initial cell attachment were three distinct tests used to investigate the effectiveness of the antibiofilm activity.

### Evaluation of the Biofilm-Forming Ability of Test Organisms

Before beginning the antibiofilm assay, the above-mentioned organisms were screened for biofilm formation using the tube method [28]. A 48 h incubation period followed the inoculation of the cultures into tubes containing 10 ml each of yeast malt broth and nutrient broth for bacteria. Media from the tubes were then discarded after which they underwent three washes in sterile phosphate-buffered saline (PBS). After that, 0.1% (v/v) crystal violet solution was used to stain the tubes. The excess discoloration was removed after 30 min by washing twice with deionized water. Following that, the bottom and wall lining revealed the development of the biofilm.

### Initial Cell Attachment Inhibition Assay

For each test microbe, in triplicate, 100 μl of the obtained compound at its concentration of (100 μg/ml) was applied to the various wells of the microtiter plate. Only microbes were utilized in the broth medium as a negative control, and the antibiotics Ciprofloxacin and Nystatin were administered to the wells in the same concentrations as the sample. Subsequently, 100 μl of the *C. albicans*, *S. aureus*, and *K. pneumoniae* cell suspensions were introduced to the wells. After 24 h, the amount of biofilm biomass that was obtained in comparison to the control, as detailed below, was used to measure the inhibitory potency of the test compound at 590 nm.

### Disruptive Ability of the Isolated Compound on Preformed Biofilms

The isolated compound's disruptive effect was tested in accordance with Onsare and Arora's method [29]. Wells were treated with 100 μl of the activated microtiter plate cultures and incubated for cell attachment to develop the biofilm. The wells with preformed biofilm were treated with 100 μl of the compound or conventional antibiotic after 24 h, and for the negative controls, the new broth was added. The wells were decanted to get rid the unattached cells before using the crystal violet test to measure the amount of biomass produced. The microtiter plate was afterwards dried in the oven for 45 min at 60°C. Subsequently, the wells were stained with 100 μl of 0.1%(w/v) crystal violet and left to develop for 15 min. Finally, the plate was rinsed with water once more, and the wells were transferred to a fresh plate after being de-stained with 125 μl of 95% ethanol. By measuring OD at 590 nm and comparing it to that of the negative control, the mass of the biofilm was determined. The following formula was used to compute the percentage of biofilm inhibition:

$$\text{\% inhibition =100-}\left[\frac{\text{OD of compound or positive control well}}{\text{OD of negative control well}}\times 100\right].$$

### Estimation of Viable Cells Using XTT

The XTT colorimetric approach, as described by Arora and Mahajan [30], was used to examine the metabolic activity of biofilms generated by organisms treated with the pure compound. The stock solution of XTT (1 mg/ml) was conducted prior to measurement. It was then filter sterilized and kept at −70°C. Then, 10 m/mol of menadione solution (Shanghai, China) was dissolved in acetone, and 2.5 ml of XTT solution was mixed with 2.5 μl and 20 μl of menadione, respectively for bacteria and yeast. As mentioned in the experiment above, the isolated compound was exposed to biofilms. After 24 h of incubation, in each well, 200 μl of menadione–XTT working solution (menadione: XTT=1:12.5) was added after the culture was removed, and rinsed three times with sterile water. The wells were then incubated for an additional 3 h at 37°C in the dark. Thereafter, a new microtiter plate was filled with 100 μl of suspension from each well, and the absorbance was recorded at 490 nm. A cell suspension that had not been exposed to the compound served as the control. Each experiment was carried out three times. The percentage viability was calculated by comparing it to the positive control.

### Anti-Proliferative Activity

The cancer cell lines employed in this study were MCF-7 (breast carcinoma), HepG-2 (liver carcinoma), and HeLa (cervical carcinoma) (Shanghai Bioleaf Technology Co. Ltd., China). According to a previous report by Yehia *et al*. [31], the cell lines were cultivated in Dulbeccós Modified Eagle Medium (DMEM; Life Technologies, USA) supplemented with 10% Fetal Bovine Serum (FBS; India), 2 mM glutamine, 100 mg/l penicillin, and 250 mg/l streptomycin (Sigma-Aldrich) utilizing 3-(4,5-dimethylthiazol-2-yl)-2,5-diphenyl tetrazolium bromide (MTT), and the isolated compound's anti-proliferative efficacy was investigated. Briefly, 100 μl of cell suspension from various cell lines was dispensed into each well of the 96-well plate at a density of 4 × 10^5^ cells/ml, and the cells were then incubated for 24 h at 37°C in a humid environment with 5% CO_2_. Cells that received no treatment served as the control. Thereafter, 100 μl of various doses (10‒250 μg/ml) of the tested compound were added to the wells and incubated for another 24 h. Following incubation, the supernatant was removed, 100 μl of MTT was added to the wells, and incubation was continued for an additional 4 h. The purple formazan crystals were subsequently dissolved in each well by adding 100 μl of dimethyl sulfoxide (DMSO), which was added after the wells had been decanted. At 570 nm, the absorbance was measured using a microplate reader (Infinite 200Pro, Tecan, Switzerland). The formula used to calculate the percentage of cell proliferation was as follows:

$$\text{\% of proliferation =}\frac{\text{Absorbance of treated wells}}{\text{Absorbance of control wells}}\times 100.$$

### DNA Topoisomerase I Relaxation Assay In Vitro for Pure Compound

The relaxation activity of DNA topoisomerase I was evaluated using agarose gel electrophoresis. Human Top I (TaKaRa, Japan) enzyme and 0.5 μg pBR322 supercoiled DNA (TaKaRa) were used in the preparation of the reaction mixture, either in the presence or absence of various quantities of pure compound (Top I: DNA Top I buffer 2 μl, DNA Top I 1 U, 0.1% bovine serum albumin 2 μl and sterile water up to 20 μl). After 30 min of incubation at 37°C, the reaction mixture was electrophoresed on 0.8% agarose gel at 80 V for 50 min with TAE running buffer, with 10-hydroxy camptothecin (CPT) serving as a positive control. Following electrophoresis, the gel was stained with ethidium bromide (1 μg/ml) and documented under UV light.

### Statistical Analysis

All investigation values are presented as mean ± SD of three independent experiments. ANOVA with Tukey's test was used to determine statistical significance. The statistical significance level was established at *p* < 0.05. SPSS v21.0 (SPSS, Inc., USA) and was utilized for statistical analysis.

## Results

### Isolation and Identification of Endophytic Fungi

In Saudi Arabia’s Eastern Region of Al Ahsa, this is most likely the first study to describe the endophytic fungi that colonize *A. sinensis* leaves. At first, isolates were identified based on spore morphology, microscopic studies and culture parameters. From leaf samples (152 segments), 124 endophytic fungal isolates representing 16 species and 8 genera (Table 1) were recovered. By analyzing the frequency of their colonization (CF), the isolates, which are related to the plant's diversity, were determined. The dominant classes were mainly Sordariomycetes with a CF of 65.8%, followed by Eurotiomycetes (29.5%), and Dothideomycetes (2.4%), all of which belong to Ascomycota. The following species of isolates were identified: one *Fusarium* species from five isolates, one *Alternaria* species from three isolates, two *Xylaria* species from three isolates, two *Colletotrichum* species from 49 isolates, four *Aspergillus* species from 23 isolates, one *Chaetomium* species from 13 isolates, two *Trichoderma* species from 12 isolates, and three *Penicillium* species from 16 isolates. The total colonization frequencies recorded were 81.6%; however, according to morphological traits, the endophytic fungus was identified as *C. acutatum*, which had a CF of 21%. The remaining isolates exhibited CFs ranging from 0.6 to 11.2% (Table 1).

From here, the richness of *C. acutatum* was found to be highest in leaves compared to other fungi. Consequently, molecular identification methods were used to confirm identity. The ITS, 28S rRNA, ACT, GAPDH and CHS sequences of *C. acutatum* were deposited in GenBank database under accession numbers ON357385, OP302756, OP501800, OP508496 and OP508497, respectively. Based on the BLAST search results with ITS and 28S rRNA sequences and morphological characters, the respective isolate was classified as *C. acutatum* and showed 100% similarity to *C. acutatum* with the ITS sequences (MN856423, MN856415, MH930414), and 28S rRNA sequences (MH877942, MH875998). In addition, based on phylogenetic analysis, sequences of ACT, GAPDH and CHS comparison showed that the taxon and other strains of *C. acutatum* have 100% identity (Fig. S2). Concatenated gene trees for the ITS, 28rRNA, ACT, GADPH, and CHS sequences showed strong support for the isolates that clustered with *C. acutatum*, and no differences were found in any of the other phylogenies that included those sequences.

In the present study, the concentrated EtOAc extract obtained from *C. acutatum* was purified by column chromatographic technique and yielded four homogenous fractions, namely R1 (80 mg), R2 (40 mg), R3 (120 mg), and R4 (75 mg). Using agar disc diffusion, each fraction was checked for antimicrobial activity against pathogenic bacterial and fungal species, and then the MIC test was performed to gain insight into the bioactive fractions for subsequent investigations. The zones of inhibition are presented in Table 2. According to the given data, R3 displayed excellent activity with zones of inhibition against *P. syringae*, *X. oryzae*, *A. hydrophila*, and *S. aureus* of 14.2, 19.8, 11.6, and 29.7 mm, respectively, while Streptomycin had inhibition zones of 33.9 mm. Meanwhile, R3 also recorded 22.8, 19.1, 27.6, and 25.4 mm against *A. flavus*. *F. solani*, *C. albicans*, and *T. rubrum*, respectively, whereas Amphotericin B, had an inhibitory zone of 33.3 mm.

Further, the MIC values were measured in vitro, as summarized in Table 2. We noticed that R3 exhibited the highest MIC value among the other fractions over all the tested pathogens, with MIC values ranging from 3.9 to 31.25 μg/ml, while Streptomycin and Amphotericin B had MIC values of 0.98 and 0.5, respectively. On the other hand, R1, R2, and R4 demonstrated moderate to little activity, with MIC values ranged from 62.5 to >1000 μg/ml.

In addition, we found that R1, R2, and R4 are ineffective against *S. aureus*, *T. rubrum*, *X. oryzae*, *A. flavus*, and *P. syringae*, while neither R1 nor R4 showed any inhibitory activity against *A. hydrophila* and *F. solani*. Taking everything into account, among all four fractions, R3 displayed consistent antimicrobial activity against the panel pathogens. This indicated that the fraction R3 has a wide range of antimicrobial activity and suggested that it might be employed to treat strains that are resistant to antibiotics. Consequently, we selected R3 for further analysis.

### Structural Identification of Isolated Compound

**Fig. 1:**
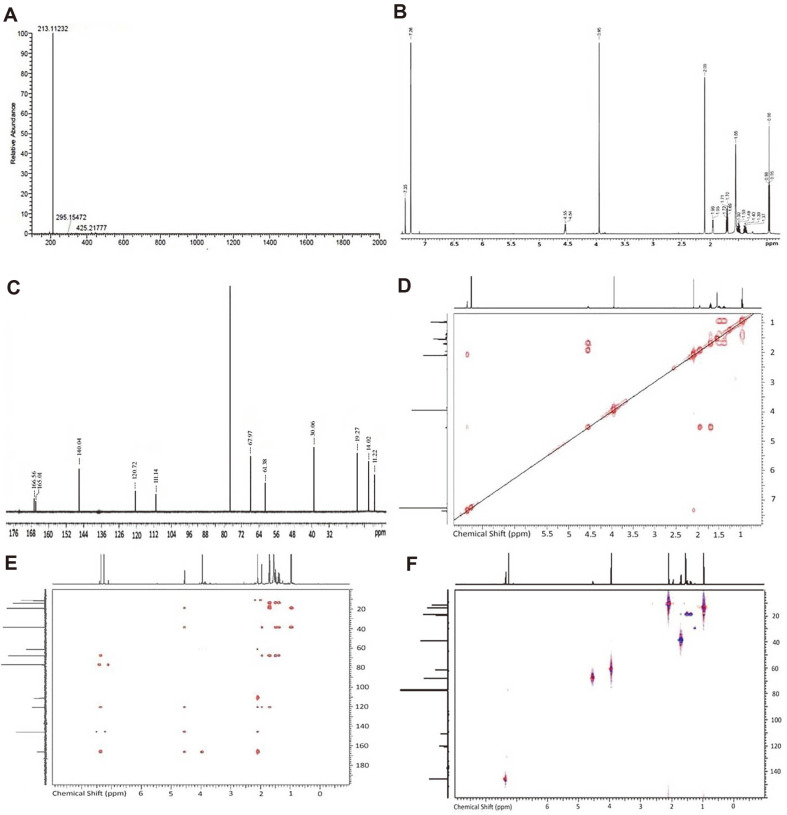
Structure elucidation: (A) HRMS, (B) ^1^H NMR, (C) ^13^C NMR, (D) COSY, (E) HMBC, (F) HSQC-edited NMR spectra of isolated of compound from *C. acutatum*.

Four fractions were obtained during the purification process using chromatographic techniques, and again the afforded fractions were screened against the above-mentioned microbes. Out of four, a fraction third (R3) exhibited extraordinary antibacterial and antifungal activity. Its characterization data is as follows: white powder (7.2 mg) and a molecular formula were assigned as C_11_H_16_O_4_ based on the signals at *m/z* = 213.11232 [M + H]+ and a second mass peak at *m/z* = 425.21777 [2M + H]+ in the HRMS (Fig. 1A), which corresponds to an Index of Hydrogen Deficiency (IHD) of four.

The substituents were identified as terminal ethyl at δ_H_ 0.98 (CH3) and δ_H_ at 1.69 (CH2), methoxy at δ_H_ 3.95, oxymethine at δ_H_ 4.55, olefinic at δ_H_ 7.35 and methyl at δ_H_ 2.09 groups in the ^1^H NMR spectrum (Fig. 1B).

In addition, the ^13^C NMR spectra (Fig. 1C) detected 11 peaks, 4 of which were quaternary carbons [δ_C_ 165.8 (C-1), δ_C_ 111.3 (C-2), δ_C_ 166.7 (C-3), and δ_C_ 120.9 (C-4)], with a methoxy peak signified by peak at δ_C_ 61.2. By using 2D-NMR, the locations of the substituents and aromatic protons were established. Figure (1) shows the three 2D-NMR experiments that were used in this study: COSY (correlation spectroscopy, ^1^H-^1^H correlations of adjacent protons, Fig. 1D), HMBC (heteronuclear multiple bond correlation, ^1^H–^13^C correlations, Fig. 1E) and HSQC-edited (heteronuclear single quantum coherence, direct ^1^H–^13^C connectivity phase edited giving results similar to DEPT-135 ^13^C NMR, Fig. 1F).

In the COSY spectrum (Fig. 1D), correlations between H-7 (δ_H_ 1.69), H-8 (δ_H_ 1.49), and H-9 (δ_H_ 0.98) were only shown in one spin system. Fig. 1E of the HMBC experiment shows a correlation between the methyl group at δ_C_ 14.0 (C-9) and the adjacent methalene carbon at δ_C_ 19.5 (C-8), as well as a second correlation for the carbon at δ_C_ 39.1 (C-7).

In the HMBC spectrum, the two methylene groups at δ_C_ 39.2 (C-7) and δ_C_ 19.5 (C-8) exhibited correlations to oxymethine at δ_C_ 68.1 (C-6). Several HMBC correlations between C-4, C-5 and H-3 were used to verify an α-pyrone ring structure. Although there were no HSQC correlations to any of the carbons in a broad singlet matching one of the protons at H-6 (H 4.66), there were HMBC correlations that helped verify the proton as belonging to the OH group (Fig. 1F). This unit was put together with the aid of certain important correlations from the olefinic proton, and the substitution patterns are supported by carbon NMR shifts.

**Fig. 2:**
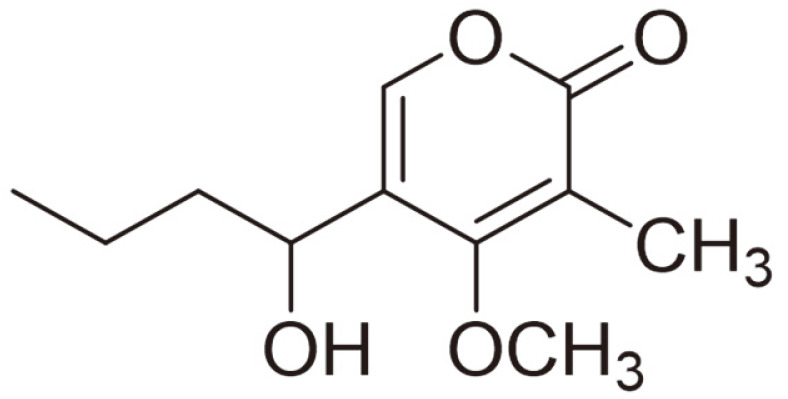
Structure of isolated compound as 5-(1-hydroxybutyl)-4-methoxy-3-methyl-2*H*-pyran-2-one, C_11_H_16_O_4_.

The chemical shift of the methyl group at this point is consistent with the presence of this subunit to complete the structure. As a result, the promising compound was identified as 5-(1-hydroxybutyl)-4-methoxy-3-methyl-2*H*-pyran-2-one (C-HMMP), and its structure is shown in Fig. 2. Interestingly, C-HMMP has not been previously isolated from *C. acutatum*.

### Antioxidant Assay

**Fig. 3:**
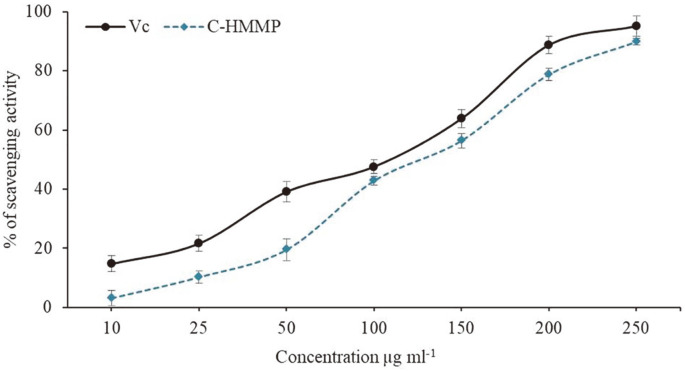
DPPH radical scavenging activity of C-HMMP produced by *C. actatum* and the standard antioxidant (Vc) at different concentrations (10‒250 μg/ml). The IC50 values of Vc and C-HMMP were determined from the equations by y = 0.3378x + 15.1018 and y = 0.3689x + 1.591, respectively. Each value is expressed as mean ± SD (*n* = 3).

The DPPH assay is an effective tool for assessing the ability of antioxidant compounds to scavenge free radicals. The dose-response curve in Fig. 3 illustrates the ability of *C. acutatum*'s C-HMMP to scavenge DPPH when compared to vitamin C. At concentrations of 10–250 μg/ml, the scavenging activity of C-HMMP on DPPH radicals was 3.1–89.9%, whereas the radical scavenging ability of the Vc (positive control) lies between 14.7–95.2%. Further, the IC50 values of C-HMMP and Vc were 131.2 and 103.3 μg/ml, respectively. The results revealed that the C-HMMP showed potential DPPH scavenging activity.

### Antimutagenic Assay

Using the Ames test, we determined whether C-HMMP would have any antimutagenic impact on the S9-dependent mutagen, 2-aminofuorene (2-AF). There was a variation in the antimutagenic inhibition rates between 6.3 and 68.6%, as shown in Table 3. The concentration of 1 mg/plate resulted in the maximum level of inhibition. Given that the amount of colonies produced by C-HMMP was comparable to that of spontaneous mode, which included no mutagens, we determined that it was neither mutagenic nor poisonous to the *S. typhimurium* strain. One-way ANOVA and Tukey's *t*-test results showed that there was a significant difference (*p* < 0.05) between the different concentrations.

### Antimalarial Assay

The C-HMMP was evaluated for antimalarial activity in vitro against the pathogenic strain *P. chabaudi* at different inhibitory doses ranging from 0.001 to 10 μg/ml (Table 4). The anti-plasmodial activity of the C-HMMP was detected after 48 h of incubation, with 17.9 to 92.3% of parasite inhibition rate and 0.15 μg/ml IC50 value.

### Antidiabetic Activity

**Fig. 4:**
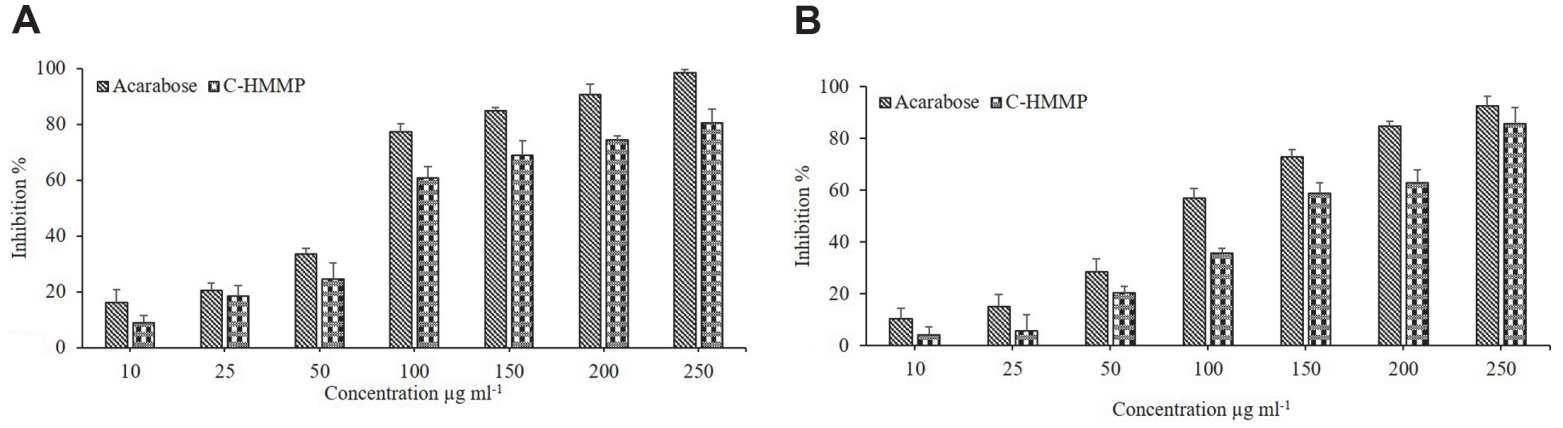
Enzymes inhibition assay of C-HMMP derived from *C. acutatum*. The percent of inhibition of α-amylase (**A**) and α-glucosidase (**B**). Acarbose was used as inhibitory agent. The values are means of three replicates (*n* = 3). Error bars in the graph represents standard deviation.

The efficacy of C-HMMP produced from *C. acutatum* to inhibit α-amylase and α-glucosidase was explored using a colorimetric assay in this investigation. According to the assay results, increasing the dose of C-HMMP results in a higher level of α-amylase and α-glucosidase inhibition percentage (Fig. 4). At the highest dose, for α-amylase inhibition (Fig. 4A), acarbose and C-HMMP presented 98.5% and 80.4%, with IC50 values of 84 and 118.6 μg/ml, respectively. Acarbose (92.4%, IC50 =108 μg/ml) and C-HMMP (85.7%, IC50 =144.7 μg/ml) both exhibit α-glucosidase inhibitory activity (Fig. 4B).

### Antibiofilm Assay

**Fig. 5:**
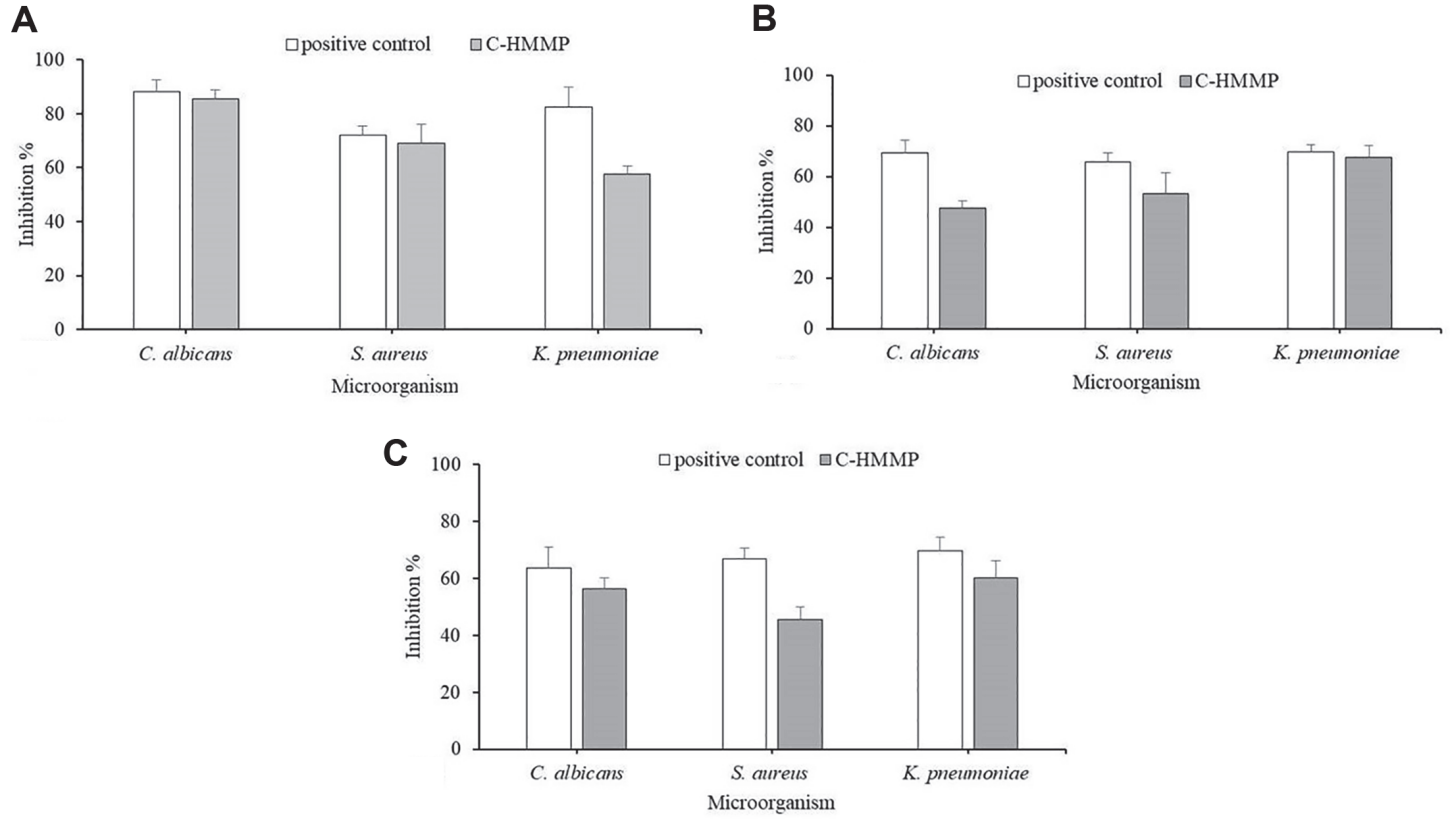
Antibiofilm activities of C-HMMP on (A) initial cell attachment, (B) mature biofilms, (C) metabolic activities of treated biofilms, measured by XTT. Different bars represent different strains, from left to right: *Candia albicans*, *Staphylococcus aureus* and *Klebsiella pneumoniae*. The bars on the graph represent mean ± SD (*n* = 3).

In our study, the ability of the potent C-HMMP to reduce biofilm was analyzed against three pathogens: *S. aureus*, *C. albicans*, and *K. pneumoniae*, which were assessed for their tendency to form biofilm. It has been shown that the antibiofilm potential exists at three distinct phases of biofilm formation investigations. As shown in Fig. 5A, the data indicated that the promising compound disrupted the biofilm-forming *C. albicans*, *S. aureus*, and *K. pneumonia*, reducing the biofilms by 72.4, 69.2, and 57.6%, respectively, at a dosage of 100 μg/ml. Nystatin and Ciprofloxacin, the reference antibiotics, simultaneously showed antibiofilm activity of 88.2, 72, and 82.6%respectively. C-HMMP was found to be the most effective towards biofilm colonization and initial cellular attachment, with no effect on strain growth.

Developed biofilms are stable, firm, and more resistant to antimicrobial agents. Upon confirmation, we noticed that C-HMMP has the capability to eliminate pre-formed biofilms, showing an inhibition percentage of 47.6%against *C. albicans*. *K. pneumonia* and *S. aureus*, on the other hand, were inhibited by 67.6% and 53.4%, respectively (Fig. 5B).

Microbial biofilms produce biofilm-essential compounds during metabolism, such as nucleic acids, proteins, and polysaccharides. Depending on the respiratory activity of the cells, the XTT test, conducted to assess metabolically active cells, shows a decrease in orange-colored formazan. The color produced is assessed colorimetrically at 490 nm and is directly proportional to the presence of living cells.

As shown in Fig. 5C, we observed that the yeast *C. albicans* showed metabolic activity inhibition of 56.4%relative to positive control (63.8%). On the other hand, *K. pneumoniae* displayed an inhibition percentage of 60.3% followed by *S. aureus* with 45.4%, while their positive control was 69.7% and 66.7%, respectively. We predict as the concentration increases, more compounds will probably be able to penetrate the mature biofilms, which would result in a marked decline in metabolic activity.

As a result, the findings indicate that the prospective compound C-HMMP reduces the ability of tested microbes to develop biofilms by inhibiting their metabolic activities.

### Cytotoxic Assay

**Fig. 6:**
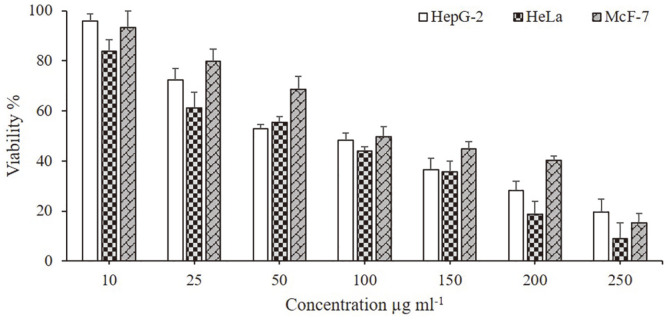
The in vitro cytotoxicity of C-HMMP on the viability of HepG-2, HeLa and MCF-7 cell lines. Tumor cells were treated with different concentrations of C-HMMP (10‒250 μg/ml), and the cell viability was evaluated by MTT assay. The data represent the IC50 values of 114.1, 90 and 133.6, respectively. The bars on the graph represent mean ± SD as a percentage of proliferation of triplicate independent experiments (*n* = 3).

Utilizing the MTT assay, C-HMMP was assayed for cytotoxic activity against three cancer cell lines; MCF-7 (breast carcinoma), HeLa (cervical carcinoma), and HepG-2 (liver carcinoma) at doses ranging from 10 to 250 μg/ml (Fig. 6). To our delight, the results revealed dose-dependent antiproliferative efficacy against all of the cancer cell lines examined. The viability of HepG-2 was decreased to 19.6% at the highest C-HMMP concentration (250 μg/ml) and the 50% inhibitory concentration (IC50) was calculated to be 114.1 μg/ml. While the viability of MCF-7 was found to be 15.3% with an IC50 value of 133.6 μg/ml, in contrast, it dropped to 8.9% in case of HeLa with an IC50 value of 90 μg/ml. This compound could be viewed as a new analog of an anticancer drug.

### DNA Topoisomerase I In Vitro Assay

**Fig. 7:**
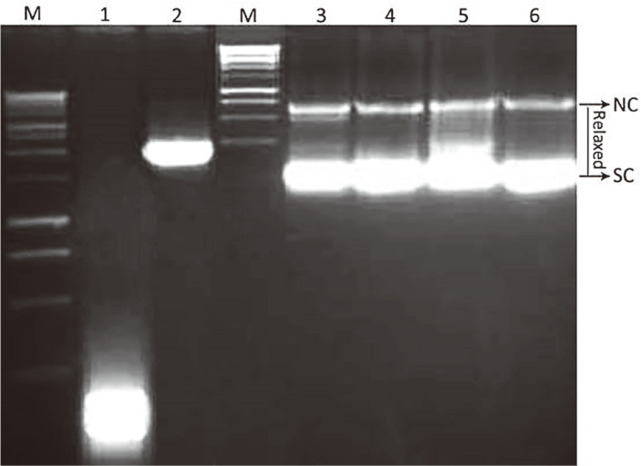
Inhibitory effects of C-HMMP and positive control (CPT) on DNA topoisomerase I. Lane 1; native supercoiled pBR322 plasmid DNA (0.5 μg) with incubation mixture in absence of Top I. Lane 2; plasmid DNA with 1U of Top I enzyme (control). Lanes 3,4 and 5; plasmid DNA with 1U of topo I in the presence of C-HMMP at concentrations 50, 150, and 250 μg/ml, respectively. Lane 6; plasmid DNA with 1U of Top I enzyme and a known DNA topoisomerase I inhibitor (CPT) at a concentration of 5 mg/ml. Negatively supercoiled pBR322 (SC), Nicked DNA (NC) and relaxed DNA (RLX) were shown.

Using the DNA from the plasmid pBR322, a plasmid-relaxing experiment was used to explore the impact of C-HMMP on topoisomerase I relaxation activity (Fig. 7). For this, 10-hydroxy camptothecin (CPT) served as a positive control for topoisomerase I inhibition. In the absence of inhibitors, the topoisomerase I was able to fully open the supercoiled DNA form (lane 2). Contrarily, CPT (lane 6) and C-HMMP blocked topoisomerase I activity in a dose-dependent manner, affecting how the supercoiled DNA unwound and resulting in a band pattern (lanes 3, 4, and 5). According to the results, a concentration of 250 μg/ml exhibited considerable DNA topoisomerase I inhibitory activity.

## Discussion

Endophytic fungi are highly taxonomically diverse and coexist in close proximity to vascular plants without transmitting disease. By encouraging the host plant’s development and disease resistance, they have substantial effects as mutualists [32]. All sections of plants have endophytic fungi, although the types and numbers of these fungi fluctuate significantly between different plants and within the same plant [33]. Season, growing stage, and various organs and tissues of the host plant are all factors that influence endophytic fungal species [34]. Moreover, by producing a variety of bioactive compounds, these endophytes can control and enhance the morphological and physiological functions of host plants under biotic and/or abiotic stress [35, 36]. This pioneering study resulted in 124 endophytic fungal isolates belonging to one phylum, 3 classes, 8 genera, and 16 morphologically different fungal species recovered from the leaves of *A. sinensis* collected from the Eastern Province, Saudi Arabia. As a result, there were numerous and diverse species of endophytic fungi. With a CF of 21%, *C. acutatum* was the most commonly isolated species. This prevalence could be attributed to the inherent properties of the fungi in these genera, which grow quickly and are extremely competitive in nonselective or plant-based media like the PDA medium utilized for isolation in this work. According to Fisher and Petrini [37], *Suaeda fruticosa* in England was endophytically populated by *C. gloeosporioides* (30% in leaves) and *Camarosporium* spp. (85.3% in stems). The fact that Saudi Arabia has a desert climate may be a contributing factor to the comparatively low overall colonization rate found in our study.

Here, we found that all isolated endophytic fungi belonged to the phylum Ascomycota, which is consistent with the findings of Petrini and Fisher [38], who reported that fungal endophytes are primarily Ascomycota. Also, Khan *et al*. [39] observed that all endophytic fungi, recovered from *Calotropis procera* and located at different sites within the Karachi University campus, belonged to the Ascomycota. Additionally, Yehia *et al*. [31] found that 82.2% of isolated endophytic fungi from *Zygophyllum mandavillei* belonged to Ascomycota. Furthermore, 350 endophytic fungal strains in total, with 98% being ascomycetes and 2% being basidiomycetes, were recovered from the seeds of *Phyllostachys edulis* and belonged to 19 different genera [40]. The most prevalent representative of an isolated endophytic population is Ascomycota [41], which validates our observation. The remaining isolated fungi, however, were identified as endophytic fungi residing in plants as symbionts and were symptomless [36, 42].

It was found that the isolated endophytic fungus from *A. sinensis* exhibited higher sequence homology with the species *C. acutatum* by searching for related ITS and 28S rRNA region sequences in GenBank. Interestingly, the multi-locus (ITS, 28S rRNA, ACT, GAPDH and CHS) phylogenetic tree analysis showed that the isolate was highly correlated to *C. acutatum*. Studies on the genus *Colletotrichum* have indicated that these fungi were abundant in the common endophytic community and might be a valuable resource for different bioactive compounds with antimicrobial [43, 44], antitumor [45], antioxidant, anti-hyperlipidemic, and anti-inflammatory [46] properties.

To overcome the challenges associated with treating infections brought on by resistant pathogens, it is critical to continue developing new antimicrobial agents. There is strong evidence that endophytes serve as a chemical reservoir for numerous bioactive compounds and are now identified as a flexible arsenal of antimicrobial drugs [43]. For instance, the endophytic fungus *Nigrospora sphaerica*, which has been demonstrated to generate bioactive substances with medicinal potential, was isolated from the leaves of *Indigofera suffruticosa* [47]. From the endophytic fungus *Berkleasmium* sp. associated with *Dioscorea zingiberensis*, nine novel Spirobisnaphthalenes were recovered, all of which have been tested for their antibacterial properties [48]. The capacity of endophytes to create biologically active secondary metabolites might contribute to a plant's therapeutic capabilities; hence, the isolation and identification of endophytic mycobiota are crucial [49]. Based on data from HRMS and comprehensive observation of the 2D NMR spectra, bioactivity-guided fractionation by column chromatography resulted in the detection of a white compound, which was designated as 5-(1-hydroxybutyl)-4-methoxy-3-methyl-2*H*-pyran-2-one (C-HMMP).

A combination of HSQC data with ^13^C- and ^1^H -NMR revealed signals attributed to one olefinic proton, one methoxy, one –OH, CH2, methoxy and methyl groups, as well as carbonyl attributed to an unsaturated lactone ring. Numerous HMBC correlations between C-4, C-5 and H3 have been employed to support the α-pyrone ring structure. The HMBC spectrum demonstrated some correlations between H7 to both of C-8 and C-9, H10 to both of C-9 and C-8, indicating the presence of a butyl side chain. Moreover, HMBC correlation between the methoxy group and C-4 validated the methoxy group's attachment. The findings of this investigation coincide with the conclusion of Masi *et al*. [50], which states that the majority of the pyrones discovered in *Colletotrichum* fungi are 2-pyrones (α-pyrones), whereas colletotrichin, azaphilone, flavonol, ergosterol, cyclohexene, and cyclopeptides are recognized *Colletotrichum* secondary metabolites [51‒55]. As far as we know, C-HMMP has never been recorded previously in a strain of this species. It is also noteworthy that the compound of interest showed remarkable antimicrobial activity against all pathogens, which could be attributed to the fact that the –OH at position C-5 strongly enhances the growth inhibitory activity [56]. Moreover, α-pyrone confirmed herein showed an outstanding action against gram-negative and gram-positive bacteria [57, 58], while it also displayed notable antifungal activity in a different investigation via disrupting the mitochondrial respiratory chain in fungi [59]. The considerable antimicrobial activity of the isolated compound compared to the other fractions implies that the bioassay-guided purification and fractionation of the fungal extract resulted in the separation of a powerful compound from *C. acutatum*.

The antioxidant activity of C-HMMP was studied utilizing a DPPH scavenging experiment. It evaluates a compound's capacity to neutralize free radicals or behave as a hydrogen donor [60, 61]. According to Pan *et al*.[62], fungal endophytes may create a variety of antioxidant compounds such as several terpenoids, flavonoids, sterols, phenols, and saponin that may neutralize DPPH free radicals. The abundance of these antioxidant agents in fruits and vegetables has significant positive health impacts, including lowering the risk of cancer, aging, heart disease, and neuro-degenerative illnesses [63].

In the present study, C-HMMP showed 3.1–89.9% scavenging potential for DPPH, and the inhibitory action increased with increasing C-HMMP doses. This is consistent with the results recorded by Uzma and Chowdappa [64] on other endophytic fungal metabolites. The decrease in the amount of DPPH molecules is related to the availability of a hydroxyl group [65]. Thus, the hydroxyl group at C-5, which is easily susceptible to proton abstraction by DPPH free radicals and forms new, more stable free radicals, may therefore be responsible for the compound's strong antioxidant activity. These stable free radicals could be stabilized via radical dispersion and delocalization [66]. Hence, C-HMMP's potent antiradical properties may play an important role as an alternative or complementary treatment for ROS-based diseases.

Our goal in the current investigation was to determine whether C-HMMP had antimutagenic properties against mutant *S. typhimurium* detected by the Ames test. The findings demonstrated a potent inhibitory impact on the S9-dependent mutagen 2-AF (polyaromatic hydrocarbon; PAH)-induced mutagenesis, and the inhibitory efficacy grew with concentration. The active metabolites of the PAHs, which are promutagens that activate to form them, covalently attach to DNA. The most active metabolizing enzyme, cytochrome p450, a member of the CYP1 family, is responsible for the creation of dihydrodiol epoxides, which may subsequently result in gene mutations [67]. In addition, 2-AF is metabolized by P-450 enzymes into a reactive carcinogenic ester, an N-hydroxyl derivative, and results in mutagenicity by interacting with DNA's guanine residues [68, 69]. Similarly, we speculated that the inhibitory action of C-HMMP on the promutagen 2-AF could be explained by its ability to interfere with or inhibit the activity of cytochrome p450-dependent enzymes. Moreover, the fact that it is a phenolic compound, which has antioxidant activity and can act as oxidation terminators by activating antioxidant enzymes or scavenging free radicals, should also be considered in relation to its structure [70].

More than 40% of people on Earth reside in regions where malaria is an endemic disease. Due to the emergence of drug-resistant forms of malaria, there is a demand for novel antimalarial medicines in many tropical nations [71]. Thus, activities of pure compounds should be categorized according to their IC50 values; a compound is characterized as very active when its IC50≤ 1 μg/ml [72, 73] in accordance with WHO recommendations and fundamental parameters for antiparasitic medication research.

Herein, with an IC50 0.15 μg/ml, C-HMMP exerted high potency against the multidrug-resistant strain of the malarial *P. chabaudi*. In contrast, Wiyakrutta [74] found that extracts of two fungal isolates from *Garcinia nigrolineata* and *G. atroviridis* demonstrated significant antimalarial activity, with IC50 values of 3.97 and 7.87 μg/ml, respectively. Studies on the relationship between structure and activity suggested that the antimalarial activity of C-HMMP might be due to its unsaturated moiety and methoxy group [75]. Interestingly, C-HMMP showed powerful anti-plasmodial activity, and hence, future examination is required to determine whether there is a link between the endophytes' habitat and their biological activities.

One of the many disorders of carbohydrate, protein, and lipid metabolism is diabetes, which affects a sizable population of individuals in undeveloped and developing nations [76]. In type 2 diabetes, the body continuously manufactures insulin, but it does not work correctly due to insulin resistance.

α-Glucosidase [E.C. 3.2.1.20] and α-amylase [E.C. 3.2.1.1] are two hydrolyzing enzymes that are crucial in the conversion of carbohydrates into sugar [76, 77]. Inhibition of these enzymes reduces glucose absorption in the small intestine, thereby lowering postprandial hyperglycemia. Moreover, inhibition of the absorption of carbohydrates from the gut is used in treating diabetes or impaired glucose tolerance. As a result, developing new sources of potent antidiabetic drugs needs time; therefore, endophytes can be a promising tool. Currently, acarbose is the drug used as a therapy for diabetes type 2, produced by *Actinoplanes* strains [78]. In the current investigation, C-HMMP exhibited significant dose-dependent inhibition of α-amylase and α-glycosidase, at the maximal dose of 250 μg/ml, C-HMMP inhibited carbohydrate hydrolyzing enzymes by more than 80%, with IC50 values of 118.6 and 84 μg/ml, respectively. Similar forms of hydrophobic couplings and hydrogen bonds were observed in the crystal structures of various inhibitor molecules and α-amylase in a previous study, suggesting that the hydroxyl group of the inhibitor improves the inhibitory activity [79]. The potential of C-HMMP identified in this study to inhibit carbohydrate-hydrolyzing enzymes might be credited to the -OH group at C-5, according to the capabilities to create quinone with the enzymés 4-oxo-pyrane structure via the -OH group. Similar to this, the endophytic fungus, *Xylariaceae* sp., which was identified from the stem of *Quercus gilva* Blume, produced a compound called 8-hydroxy-6,7-dimethoxy-3-methylisocoumarine, which was recovered and characterized. This compound demonstrated high α-glycosidase inhibitory activity [80]. Additionally, antioxidant compounds are essential in reducing the risk of oxidative stress-related disorders, such as diabetes, cancer, and cardiovascular disease [81]. Thus, lowering oxidative stress will prevent the development of diabetes [82]. In this investigation, it very well may be reasoned that C-HMMP restrained the activity of α-glucosidase and α-amylase.

Microorganisms create a complex relationship called a biofilm on surrounding tissues that is difficult to break up. It is a significant threat and one method of resistance. Pathogens can resist standard medications by a variety of mechanisms, including the encoding of multidrug efflux pumps, the reduction of membrane permeability, the formation of biofilms, and the inactivation of cell membrane receptors [83]. Effective ways to render pathogens in biofilm more susceptible to antibiotics and host immune systems has been suggested [84], with examples including chemical reactions that prevent the synthesis of the biofilm matrix, enzymes that dissolve the biofilm's matrix polymers, and analogs of microbial signaling molecules that obstruct cell-to-cell interaction, which is crucial for normal biofilm formation [85].

Finding an appropriate antibiotic is one technique for addressing the issue of resistant microbial pathogens, and this focus on endophytes has led scientists to seek out and create novel antibiofilm drugs. In the present study, C-HMMP exhibited remarkable antibiofilm action. The effectiveness of C-HMMP against *C. albicans* during initial cell attachment is nearly identical to that of the positive control, Nystatin. Furthermore, against *S. aureus* and *K. pneumonia*, C-HMMP showed an excellent inhibition rate of 69.2%, and 57.6%, respectively. Bacterial biofilm development is significantly influenced by cell surfaces and cell-cell interactions [86]. These data suggested that C-HMMP inhibited or disrupted cell-cell interactions that generated antibiofilm actions. Further, the compound's antimicrobial activity may explain why this compound can eradicate biofilms; strong antibiofilm activity is a characteristic that is frequently linked to drugs that disturb membranes [87].

Commercial drugs including amphotericin B, itraconazole, and fluconazole, which are far less effective against biofilms, find it challenging to eliminate mature biofilms. Here, C-HMMP exhibited a clear disruptive potential against pre-formed biofilms with inhibition ranging from 45–60% after treatment with 100 μg/ml of C-HMMP. This indicates that C-HMMP effectively lowers the secretion of the extracellular matrix, affects the biofilm structure, and is able to operate on cells that were coiled by the extracellular matrix. Further analysis found that C-HMMP was capable of rupturing the mature biofilm by inhibiting the cell aggregate formation of *C. albicans*, *S. aureus*, and *K. pneumonia*. This is because our compound has a hydroxyl group at position C-5, which makes the entire molecule more hydrophilic and facilitates penetration into the extracellular polymeric substance of the biofilm to reach bacterial cells [88]. Thus, the reduction in biofilm amount is caused by the inability to create the biofilm rather than by the prevention of bacterial or yeast growth. According to Rajivgandhi *et al*. [89], a crude product (100 μg/ml) isolated from endophytic actinomycetes displayed antibiofilm action, inhibiting the growth of *K. pneumoniae* and *P. aeruginosa* bacterial films by 77% and 82%, respectively.

In addition, by colorimetrically quantifying XTT reduction, we were able to evaluate the impact of C-HMMP on metabolic activity [90]. Numerous comparisons with NCCLS standard susceptibility tests have already shown the validity of this procedure, which has begun to gain significant use [83]. The quick turnaround time for results is one of this method's key benefits. It can be time-consuming to evaluate the antibiotic susceptibility of cells in biofilms; hence, using a trustworthy and efficient approach is preferred in a clinical laboratory. To summarize, our data suggest that C-HMMP plays an important role in regulating biofilm adherence, anchoring, and matrix formation. This could serve as a therapeutic potential for treating infections related to the above strains.

The demand to create new chemotherapeutic drugs is expanding due to the risk of multi-drug resistance (MDR), as well as the unpleasant side effects and high expense of chemotherapy. Endophytes have been the subject of numerous studies to find innovative and potent cancer treatments [91, 92]. With IC50 values of 133.6, 114.1, and 90 μg/ml, respectively, C-HMMP exhibited different degrees of antiproliferative activity against the MCF-7, HepG-2, and HeLa cell lines in the current investigation.

Undoubtedly, the hydroxyl group is important as well as the 2-pyrone subunit which plays an important role in understanding mechanism of action and the correlations between structure and activity [93]. Moreover, the methoxylation performed effectively for the antiproliferative action in vitro [94]. In previous studies, cytotoxic activity against cancer cell lines such as Ovarian and Colorectal [95], HeLa [96], and CML [97] has been documented on a variety of 2-pyrone natural and synthesized compounds. These results contribute to the evidence that C-HMMP has relevant anticancer activity in vitro, indicating the possibility for using this type of compound as a precursor for the creation of novel anticancer drugs.

Existing anticancer drugs have been observed to be both highly hazardous and selectively ineffective. Thus, many researchers frequently discover brand new possible targets that are particular to or selective of cancer cells [98]. Many drugs that have been utilized to treat cancer have DNA as their molecular target [99]. By controlling the topological configuration of DNA, DNA topoisomerases are crucial for biological activities such as DNA replication, recombination, transcription, segregation, and chromosomal assembly [100, 101]. Several potent anticancer drugs have been reported to target eukaryotic DNA topoisomerase I, for instance, camptothecin [102, 103]. To explore the suppression of DNA topoisomerase I activity, several researchers have used the relaxation assay, which in this work uses a supercoiled plasmid as substrate. In ethidium bromide stained gels, the relaxed isomers travel more slowly than the supercoiled isomers, making it simple to discriminate between the supercoiled substrate and its relaxed product. Since more compact molecules move more quickly than their more relaxed counterparts, it is possible to distinguish between changes in molecular form without a corresponding change in molecular weight [104].

At the end of the electrophoresis, different bands would be formed if topoisomerases totally relaxed the DNA molecules and there was an equilibrium between the multiple topological forms of the DNA molecules. On the other hand, a faster-moving single band would be obtained if the C-HMMP blocked the catalytic activities of topoisomerases and as a result, all the DNA molecules were in a supercoiled shape. The current findings support potential for the design and development of novel chemotherapeutic drugs in the future because DNA topoisomerases are crucial targets for cancer treatment.

Finally, it seems that the advantageous biological impact of the ring-linked methoxy group in C-HMMP, which has been verified, can be prudently explored in the future design of a new series of analogs, as well as coupled with the effect of the –OH group at C-5 of the lactone ring, as observed for γ-pyrone, in the design of novel analogs in further studies.

The current findings might support the idea that endophytes are being explored as a promising source for new bioactive compounds [7]. The results presented in this research showed that C-HMMP of the endophytic fungus *C. acutatum* from leaves of *A. sinensis* has antimicrobial, antioxidant, antibiofilm, antimalarial, antimutagenic, and antiproliferative activities.

Thus, *C. acutatum* is crucial in the search for bioactive metabolites and may serve as an alternate source for the creation of therapeutic agents that are difficult to produce through chemical synthesis. This investigation, however, will serve as a precursor for additional extensive studies on the biology of endophyte bioactive natural compounds. Future research is required to pinpoint the exact mechanism of action and determine how this compound behaves when taken in accordance with the traditional healers' dose recommendations.

## Supplemental Materials

Supplementary data for this paper are available on-line only at http://jmb.or.kr.

## Figures and Tables

**Table 1:** Isolation, colonialization and dominance frequency of endophytic fungi from plant leaf of *Angelica sinensis*.

Endophytic fungi		No.	*CF%*	**DF%

Genus	Species			
*Penicillium*		**16**		
	*P. polnicum*	4	2.6	3.2
	*P. citrinum*	9	5.9	7.2
	*P. chrysogenum*	3	2.0	2.4
*Trichoderma*		**12**		
	*T. harzianum*	8	5.3	6.5
	*T. viridae*	4	2.6	3.2
*Chaetomium*		**13**		
	*C. globossum*	13	8.5	10.4
*Aspergillus*		**23**		
	*A. ochraceous*	2	1.3	1.6
	*A. terreus*	1	0.6	0.7
	*A. flavus*	7	4.6	5.6
	*A. niger*	11	7.2	8.8
*Colletotrichum*		**49**		
	*C. gloeosporioides*	17	11.2	13.7
	*C. acutatum*	32	21.0	25.7
*Xylaria*		**3**		
	*X. berteri*	1	0.6	0.7
	*X. laevis*	2	1.3	1.6
*Alternaria*		**3**		
	*Alt. macrospora*	3	2.0	2.4
*Fusarium*		**5**		
	*F. oxysporum*	5	3.3	4.0
Total		**124**	81.6	

*CF%, colonialization frequency percentage; **DF%, fungal dominance frequency percentage.

**Table 2:** Bio-guided antimicrobial activities of endophytic fungal-fractions.

Strain	Fraction no.							

	R1		R2		R3		R4	

	IZ	MIC	IZ	MIC	IZ	MIC	IZ	MIC
*Pseudomonas syringae*	5.7±0.47	500	2.1 ± 0.41	500	14.2±0.5	15.62	na	>1000
*Xanthomonas oryzae*	3.5±0.40	125	na	>1000	19.8±0.20	7.81	4.2±0.45	500
*Aeromonas hydrophila*	na	>1000	3.7±0.88	125	11.6±0.70	3.9	na	>1000
*Staphylococcus aureus*	na	>1000	6.1±0.66	31.25	29.7±0.28	7.81	8.9±0.22	125
*Streptomycin*	35.3±0.85	0.25	28.8±0.48	0.25	33.9±0.49	0.98	30.0±0.87	0.25
*Aspergillus flavus*	10.5±0.22	250	na	>1000	22.8±0.11	15.62	10.2±0.78	62.5
*Fusarium solani*	na	>1000	na	>1000	19.1±0.79	31.25	na	>1000
*Candida albicans*	2.0±0.60	62.5	5.9±0.50	125	27.6±0.55	3.9	4.2±0.70	250
*Trichophyton rubrum*	na	>1000	3.0±0.92	500	25.4±0.40	1.95	2.8±0.96	62.5
Amphotericin B	22.2±0.97	0.25	24.8±0.40	0.25	33.3±0.31	0.5	31.1±0.80	0.5
DMSO	–	–	–	–	–	–	–	–

IZ: Growth Inhibition Zone (mm) ± SD; MIC: Minimum inhibitory concentration; na: not active Amphotericin B and Streptomycin as antifungal and antibacterial positive control, respectively.

1% dimethyl sulphoxide (DMSO); negative control.

**Table 3:** Antimutagenic effect of C-HMMP produced by *C. acutatum* on the mutagenicity induced by S9-dependent mutagen (2-AF).

Conc. (mg/plate)	No. of revertant colonies	% of inhibition
0.05	867 ± 4.5^e^	6.3
0.1	752 ± 11.1^d^	19.5
0.25	586 ± 8.4^c^	38.4
0.5	465 ± 5.2^b^	52.2
1.0	321 ± 3.3^a^	68.6
Positive control (2-AF)	923 ± 3.7	−
Negative control (without mutagen)	43 ± 1.4	−
Spontaneous	46 ± 2.3	−

Values are given, as mean ± SE. Different letters between the columns are significantly different (Tukey’s test, *p* ≤ 0.05).

**Table 4:** In vitro antimalarial activity of C-HMMP against *P. chabaudi* after 48 h incubation period.

Conc. (μg/ml)	% of inhibition
0.001	17.9
0.01	44.7
0.1	57.6
1.0	85.5
10	92.3
Negative control (without mutagen)	−

The concentration of C-HMMP that inhibits parasite growth by 50% was determined; IC50= 0.015 μg/ml. Antiplasmodial activity of C-HMMP was classified according to its IC50 value as high (IC50 ≤1 μg/ml) [73].

## References

[1] Koehn FE, Carter GT (2005). The evolving role of natural products in drug discovery. Nat. Rev. Drug Discov..

[2] Clardy J, Walsh C (2004). Lessons from natural molecules. Nature.

[3] Schulz B, Boyle C, Draeger S, Römmert AK, Krohn K (2002). Endophytic fungi: a source of novel biologically active secondary metabolites. Mycol. Res..

[4] Saikkonen K, Faeth SH, Helander M, Sullivan T (1998). Fungal endophytes: a continuum of interactions with host plants. Annu. Rev. Ecol. Syst..

[5] Aly AH, Debbab A, Kjer J, Proksch P (2010). Fungal endophytes from higher plants: a prolific source of phytochemicals and other bioactive natural products. Fungal Divers.

[6] Kaul S, Gupta S, Ahmed M, Dhar MK (2012). Endophytic fungi from medicinal plants: a treasure hunt for bioactive metabolites. Phytochem. Rev..

[7] Strobel GA (2003). Endophytes as sources of bioactive products. Microbes Infect..

[8] Kogel KH, Franken P, Hückelhoven R (2006). Endophyte or parasite - what decides?. Curr. Opin. Plant Biol..

[9] Porras-Alfaro A, Bayman P (2011). Hidden fungi, emergent properties: endophytes and microbiomes. Annu. Rev. Phytopathol..

[10] Chutulo EC, Chalannavar RK (2018). Endophytic mycoflora and their bioactive compounds from *Azadirachta indica*: a comprehensive review. J. Fungi.

[11] Kharwar RN, Mishra A, Gond SK, Stierle A, Stierle D (2011). Anticancer compounds derived from fungal endophytes: their importance and future challenges. Nat. Prod. Rep..

[12] Barnett HL, Hunter BB (1998). Illustrated Genera of Imperfect Fungi, (No. Ed. 4).

[13] Cooke WB (1958). The ecology of the fungi. Bot. Rev..

[14] El-Shafie AK (1996). Soil fungi in Qatar and other Arab countries. Econ. Bot..

[15] Suryanarayanan TS, Murali TS, Venkatesan G (2003). Endophytic fungal communities in leaves of tropical forest trees: diversity and distribution patterns. Curr. Sci..

[16] White TJ, Bruns T, Lee S, Taylor J, Innis MA, Gelfand DH, Sninsky JJ, White TJ (1990). Amplification and direct sequencing of fungal ribosomal RNA genes for phylogenetics. PCR Protocols.

[17] Vilgalys R, Hester M (1990). Rapid genetic identification and mapping of enzymatically amplified ribosomal DNA from several Cryptococcus species. J. Bacteriol..

[18] Carbone I, Kohn LM (1999). A method for designing primer sets for speciation studies in filamentous ascomycetes. Mycologia.

[19] Guerber JC, Liu B, Correll JC, Johnston PR (2003). Characterization of diversity in *Colletotrichum acutatum* by sequence analysis of two gene introns, mtDNA and intron RFLPs, and mating compatibility. Mycologia.

[20] CLSI (2015). Performance standards for antimicrobial disk susceptibility test; approved standard-Twelfth Edition.

[21] CLSI (2010). Method for antifungal disk diffusion susceptibility testing of non dermatophyte filamentous fungi; approved guideline.

[22] CLSI (2008). Reference Method for Broth Dilution Antifungal Susceptibility Testing of Filamentous Fungi; Approved Standard: CLSI Document M38-A2, 2nd Edn.

[23] Zhao Y, Du SK, Wang H, Cai M (2014). In vitro antioxidant activity of extracts from common legumes. Food Chem..

[24] Maron D, Ames BN (1983). Revised methods for the *Salmonella* mutagenicity test. Mutat. Res..

[25] Budimulya AS, Syafruddin Tapchaisri P, Wilariat P, Marzuki S (1997). The sensitivity of *Plasmodium* protein synthesis to prokaryotic ribosomal inhibitors. Mol. Biochem. Parasitol..

[26] Worthington TM (1982). Enzymes and Related Biochemicals. Biochemical Products Division.

[27] Zhang J, Zhao S, Yin P, Yan L, Han J, Shi L (2014). α-Glucosidase inhibitory activity of polyphenols from the burs of *Castanea mollissima* blume. Molecules.

[28] Christensen GD, Simpson WA, Bisno AL, Beachey EH (1982). Adherence of slime-producing strains of *Staphylococcus epidermidis* to smooth surfaces. Infec. Immun..

[29] Onsare JG, Arora DS (2015). Antibiofilm potential of flavonoids extracted from *Moringa oleifera* seed coat against *Staphylococcus aureus*, *Pseudomonas aeruginosa* and *Candida albicans*. J. Appl. Microbiol..

[30] Arora DS, Mahajan H (2019). Major phytoconstituents of Prunus cerasoides responsible for antimicrobial and antibiofilm potential against some reference strains of pathogenic bacteria and clinical isolates of MRSA. Appl. Biochem. Biotechnol..

[31] Yehia RS, Osman GH, Assaggaf H, Salem R, Mohamed MS (2020). Isolation of potential antimicrobial metabolites from endophytic fungus *Cladosporium cladosporioides* from endemic plant *Zygophyllum mandavillei*. S. Afr. J. Bot..

[32] Kaur T, Hassanuzaman M, Filho MCM, Fujita M, Nogueira TAR (2020). Fungal endophyte-host plant interactions: role in sustainable agriculture, in Sustainable Crop Production.

[33] Shan TJ, Feng H, Xie Y, Shao C, Wang J, Mao ZL (2019). Endophytic fungi isolated from *Eucalyptus citriodora* Hook. f. and antibacterial activity of crude extracts. Plant Prot..

[34] Miguel PSB, Delvaux JC, Oliveira MNV, Moreira BC, Borges AC, Totola MR (2017). Diversity and distribution of the endophytic fungal community in eucalyptus leaves. Afr. J. Microbiol. Res..

[35] Gouda S, Das G, Sen SK, Shin HS, Patra JK (2016). Endophytes: a treasure house of bioactive compounds of medicinal importance. Front. Microbiol..

[36] Shah S, Shrestha R, Maharjan S, Selosse MA, Pant B (2019). Isolation and characterization of plant growth-promoting endophytic fungi from the roots of *Dendrobium moniliforme*. Plants.

[37] Fisher PJ, Petrini O (1987). Location of fungal endophytes in tissues of *Suaeda fruticosa*: a preliminary study. Transact. Brit. Mycol. Soc..

[38] Petrini O, Fisher PJ (1986). Fungal endophytes in *Salicornia perennis*. Transact. Brit. Mycol. Soc..

[39] Khan R, Shahzad S, Choudhary M, Khan SA, Ahmad A (2007). Biodiversity of endophytic fungi isolated from *Calotropis procera* (Ait.) R. Br.. Pak. J. Bot..

[40] Shen XY, Cheng YL, Cai CJ, Fan L, Gao J, Hou CL (2014). Diversity and antimicrobial activity of culturable endophytic fungi isolated from moso bamboo seeds. PLoS One.

[41] Koukol O, Kolařík M, Kolářová Z, Baldrian P (2012). Diversity of foliar endophytes in wind-fallen *Picea abies* trees. Fungal Divers.

[42] Hamzah TNT, Lee SY, Hidayat A, Terhem R, Faridah-Hanum I, Mohamed R (2018). Diversity and characterization of endophytic fungi isolated from the tropical mangrove species, *Rhizophora mucronata*, and identification of potential antagonists against the soil-borne fungus, *Fusarium solani*. Front. Microbiol..

[43] Arivudainambi USE, Anand TD, Shanmugaiah V, Karunakaran C, Rajenrdan A (2011). Novel bioactive metabolites producing endophytic fungus *Colletotrichum gloeosporioides* against multidrug resistant *Staphylococcus aureus*. FEMS Immunol. Med. Microbiol..

[44] Zou WX, Meng JC, Lu H, Chen GX, Shi GX, Zhang TY (2000). Metabolites of *Colletotrichum gloeosporioides*, an endophytic fungus in *Artemisia mongolica*. J. Nat. Prod..

[45] Xiong ZQ, Yang YY, Zhao N, Wang Y (2013). Diversity of endophytic fungi and screening of fungal paclitaxel producer from *Anglojap yew*, Taxus x media. BMC Microbiol..

[46] Zhang Q, Wei X, Wang J (2012). Phillyrin produced by *Colletotrichum gloeosporioides*, an endophytic fungus isolated from *Forsythia suspensa*. Fitoterapia.

[47] dos Santos IP, da Silva LCN, da Silva MV, de Araújo JM, Cavalcanti MD, Lima VLD (2015). Antibacterial activity of endophytic fungi from leaves of *Indigofera suffruticosa* Miller (Fabaceae). Front. Microbiol..

[48] Shan TJ, Tian J, Wang XH, Mou Y, Mao ZL, Lai DW (2014). Bioactive spirobisnaphthalenes from the endophytic fungus *Berkleasmium* sp. J. Nat. prod..

[49] Kusari S, Pandey SP, Spiteller M (2013). Untapped mutualistic paradigms linking host plant and endophytic fungal production of similar bioactive secondary metabolites. Phytochemistry.

[50] Masi M, Cimmino A, Boari A, Tuzi A, Zonno MC, Baroncelli R (2017). Colletochlorins E and F, new phytotoxic tetrasubstituted pyran-2-one and dihydrobenzofuran, isolated from *Colletotrichum higginsianum* with potential herbicidal activity. J. Agric. Food Chem..

[51] Garcia-Pajon CM, Collado IG (2003). Secondary metabolites isolated from *Colletotrichum* species. Nat. Prod. Rep..

[52] Gohbara M, Kosuge Y, Yamasaki S, Kimura Y, Suzuki A, Tamura S (1978). Isolation, structures and biological activities of colletotrichins, phytotoxic substances from *Colletotrichum nicotianae*. Agric. Biol. Chem..

[53] Liu HX, Tan HB, Chen YC, Li SN, Li HH, Zhang WM (2018). Secondary metabolites from the *Colletotrichum gloeosporioides* A12, an endophytic fungus derived from *Aquilaria sinensis*. Nat. Prod. Res..

[54] Lu H, Zou WX, Meng JC, Hu J, Tan RX (2000). New bioactive metabolites produced by *Colletotrichum* sp., an endophytic fungus in *Artemisia annua*. Plant Sci..

[55] Wang WX, Kusari S, Laatsch H, Golz C, Kusari P, Strohmann C (2016). Antibacterial Azaphilones from an endophytic fungus, *Colletotrichum* sp.BS4. J. Nat. Prod..

[56] Huang L, Luo H, Li Q, Wang D, Zhang J, Hao X (2015). Pentacyclic triterpene derivatives possessing polyhydroxyl ring A inhibit Gram-positive bacteria growth by regulating metabolism and virulence genes expression. Eur. J. Med. Chem..

[57] Rios JL, Recio MC, Villar A (1991). Isolation and identification of the antibacterial compounds from *Helichrysum stoechas*. J. Ethnopharmacol..

[58] Zhu H, Li D, Yan Q, An Y, Huo X, Zhang T (2019). α-Pyrones, secondary metabolites from fungus *Cephalotrichum microsporum* and their bioactivities. Bioorg. Chem..

[59] Tomás-Lorente F, Iniesta-Sanmartín E, Tomás-Barberán FA, Trowitzsch-Kienast W, Wray V (1989). Antifungal phloroglucinol derivatives and lipophilic flavonoids from *Helichrysum decumbens*. Phytochemistry.

[60] Da Porto C, Calligaris S, Celotti E, Nicoli MC (2000). Antiradical properties of commercial cognacs assessed by the DPPH(.) test. J. Agric. Food Chem..

[61] Soare JR, Dinis TC, Cunha AP, Almeida LM (1997). Antioxidant activities of some extracts of *Thymus zygis*. Free Radic. Res..

[62] Pan F, Su TJ, Cai SM, Wu W (2017). Fungal endophyte-derived *Fritillaria unibracteata var. wabuensis*: diversity, antioxidant capacities in vitro and relations to phenolic, flavonoid or saponin compounds. Sci. Rep..

[63] Baxter A, Mittler R, Suzuki N (2013). ROS as key players in plant stress signaling. J. Exp. Bot..

[64] Uzma F, Chowdappa S (2017). Antimicrobial and antioxidant potential of endophytic fungi isolated from ethnomedicinal plants of Western Ghats Karnataka. J. Pure Appl. Microbiol..

[65] Brunetti C, Martina D, Ferdinando MD, Fini A, Pollastri S, Tattini M (2013). Flavonoids as antioxidants and developmental regulators: relative significance in plants and humans. Int. J. Mol. Sci..

[66] Gherraf N, Segni L, Brahim L, Samir H (2011). Evaluation of antioxidant potential of various extract of *Traganum nudatum* Del. Plant Sci. Feed.

[67] Kondraganti SR, Fernandez-Salguero P, Gonzalez FJ, Ramos KS, Jiang W, Moorthy B (2003). Polycyclic aromatic hydrocarbon inducible DNA adducts: evidence by 32P-postlabeling and use of knockout mice for Ah receptor-independent mechanisms of metabolic activation in vivo. Int. J. Cancer.

[68] DeBaun JR, Smith JY, Miller EC, Miller JA (1970). Reactivity in vivo of the carcinogen *N*-hydroxy-2-acetylaminofuorene: increase by sulfate ion. Science.

[69] Miller JA (1970). Carcinogenesis by chemicals: an overview-GHA clowes memorial lecture. Cancer Res..

[70] Phadungkit M, Somdee T, Kangsadalampai K (2012). Phytochemical screening, antioxidant and antimutagenic activities of selected Thai edible plant extracts. J. Med. Plants Res..

[71] Cowman AF, Duraisingh MT (2001). An old enemy, a new battle plan: perspectives on combating drug-resistance malaria. EMBO Rep..

[72] Pink R, Hudson A, Mouriès MA, Bendig M (2005). Opportunities and challenges in antiparasitic drug discovery. Nat. Rev. Drug Discov..

[73] Jansen O, Tits M, Angenot L (2012). Anti-plasmodial activity of *Dicoma tomentosa* (Asteraceae) and identification of urospermal A-15-O-acetate as the main active compound. Malar. J..

[74] Wiyakrutta S, Sriubolmas N, Panphut W, Thongon N, Danwisetkanjana K, Ruangrungsi N (2004). Endophytic fungi with antimicrobial, anti-cancer and antimalarial activities isolated from Thai medicinal plants. World J. Microbiol. Biotechnol..

[75] Jiménez-Romero C, Ortega-Barría E, Arnold AE, Cubilla-Rios L (2008). Activity against *Plasmodium falciparum* of lactones isolated from the endophytic fungus *Xylaria* sp. Pharm. Biol..

[76] Surya S, Salam AD, Tomy DV, Carla B, Kumar RA, Sunil C (2014). Diabetes mellitus and medicinal plasnts-a review. Asian Pac. J. Trop. Dis..

[77] Wu PP, Zhang K, Lu YJ, He P, Zhao SQ (2014). In vitro and in vivo evaluation of the antidiabetic activity of ursolic acid derivatives. Eur. J. Med. Chem..

[78] Schmidit D, Frommer W, Junge B, Muller L, Wingender W, Truscheit E (1977). α-Glucosidase inhibitors. Naturwissenschaften.

[79] Sohretoglu D, Sari S, Barut B, Özel A (2018). Discovery of potent α-glucosidase inhibitor favonols: insights into mechanism of action through inhibition kinetics and docking simulations. Bioorg. Chem..

[80] Indrianingsih AW, Tachibana S (2017). α-Glucosidase inhibitor produced by an endophytic fungus, *Xylariaceae* sp. QGS 01 from *Quercus gilva* Blume. Food Sci. Human Wellness.

[81] Wu XJ, Hansen C (2008). Antioxidant capacity, phenolic content, and polysaccharide content of *Lentinus edodes* grown in whey permeate‐based submerged culture. J. Food Sci..

[82] Burton GW, Ingold KU, J. Miquel (1999). Mechanism of antioxidant action: preventive and chain breaking antioxidants. CRC handbook of free radicals and antioxidants in biomedicine (Chap. 10, pp. 29-43).

[83] Baral B, Mozafari MR (2020). Strategic moves of "superbugs" against available chemical scaffolds: signaling, regulation, and challenges. ACS Pharmacol. Transl. Sci..

[84] Stewart PS, Costerton JW (2001). Antibiotic resistance of bacteria in biofilms. Lancet.

[85] Nemoto K, Hirota K, Ono T, Murakami K, Murakami K, Nagao D (2000). Effect of Varidase (streptokinase) on biofilm formed by *Staphylococcus aureus*. Chemotherapy.

[86] O'Toole G, Kaplan HB, Kolter R (2000). Biofilm formation as microbial development. Annu. Rev. Microbiol..

[87] Hurdle JG, O'Neill AJ, Chopra I, Lee RE (2011). Targeting bacterial membrane function: an underexploited mechanism for treating persistent infections. Nat. Rev. Microbiol..

[88] Wojnicz D, Tichaczek-Goska D, Kicia M (2015). Pentacyclic triterpenes combined with ciprofloxacin help to eradicate the biofilm formed in vitro by *Escherichia coli*. Indian J. Med. Res..

[89] Rajivgandhi G, Vijayan R, Maruthupandy M, Vaseeharan B, Manoharan N (2018). Antibiofilm effect of *Nocardiopsis* sp. GRG 1 (KT235640) compound against biofilm forming Gram negative bacteria on UTIs. Microb. Pathog..

[90] Tunney M, Ramage G, Field T (2004). Rapid colorimetric assay for antimicrobial susceptibility testing of *Pseudomonas aeruginosa*. Antimicrob. Agents Chemother..

[91] Nascimento AM, Conti R, Turatti IC, Cavalcanti BC, Costa-Lotufo LV, Pessoa C (2012). Bioactive extracts and chemical constituents of two endophytic strains of *Fusarium oxysporum*. Rev. Bras. Farmacogn..

[92] Zhan J, Burns AM, Liu MX, Faeth SH, Gunatilaka AA (2007). Search for cell motility and angiogenesis inhibitors with potential anticancer activity: Beauvericin and other constituents of two endophytic strains of *Fusarium oxysporum*. J. Nat. Prod..

[93] Fairlamb IJS, Marrison LR, Dickinson JM, Lu FJ, Schmidt JP (2004). 2-Pyrones possessing antimicrobial and cytotoxic activities. Bioorg. Med. Chem..

[94] Barcelos RC, Pastre JC, Caixeta V, Vendramini-Costa DB, de Carvalho JE, Pilli RA (2012). Synthesis of methoxylated goniothalamin, aza-goniothalamin and γ-pyrones and their in vitro evaluation against human cancer cells. Bioorg. Med. Chem..

[95] Suzuki K, Kuwahara A, Yoshida H, Fujita SI, Nishikiori T, Nakagawa T (1997). NF00659A(1), A(2), A(3), B-1 and B-2, novel antitumor antibiotics produced by *Aspergillus* sp. NF00659 .1. Taxonomy, fermentation, isolation and biological activities. J. Antibiot..

[96] Kondoh M, Usui T, Kobayashi S, Tsuchiya K, Nishikawa K, Nishikiori T (1998). Cell cycle arrest and antitumor activity of pironetin and its derivatives. Cancer Lett..

[97] Marrison LR, Dickinson JM, Fairlamb IJS (2002). Bioactive 4-substituted-6-methyl-2-pyrones with promising cytotoxicity against A2780 and K562 cell lines. Bioorg. Med. Chem. Lett..

[98] Kohn KW (1996). DNA filter elution: a window on DNA damage in mammalian cells. Bioessays.

[99] Hurley LH (2002). DNA and associated processes as targets for cancer therapy. Nat. Rev. Cancer.

[100] Nitiss JL (1998). Investigating the biological functions of DNA topoisomerases in eukaryotic cells. Biochim. Biophys. Acta..

[101] Wang JC (1996). DNA topoisomerases. Annu. Rev. Biochem..

[102] Liu LF (1989). DNA topoisomerase poisons as antitumor drugs. Annu. Rev. Biochem..

[103] Pommier Y (1998). Diversity of DNA topoisomerases I and inhibitors. Biochimie.

[104] Barrett JF, Sutcliffe JA, Gootz TD (1990). In vitro assays used to measure the activity of topoisomerases. Antimicrob. Agents Chemother..

