# Supplementary material for: Multi-Function of a New Bioactive Secondary Metabolite Derived from Endophytic Fungus Colletotrichum acutatum of Angelica sinensis
Source: J Microbiol Biotechnol. 2022 Dec 27;33(6):806–22. doi: 10.4014/jmb.2206.06010 (PMC10333465; doi:10.4014/jmb.2206.06010)
Supplement: Supplementary file 1 [file jmb-33-6-806-supple.pdf]

J. Microbiol. Biotechnol.
https://doi.org/10.4014/jmb.2206.06010

Supplementary Figures

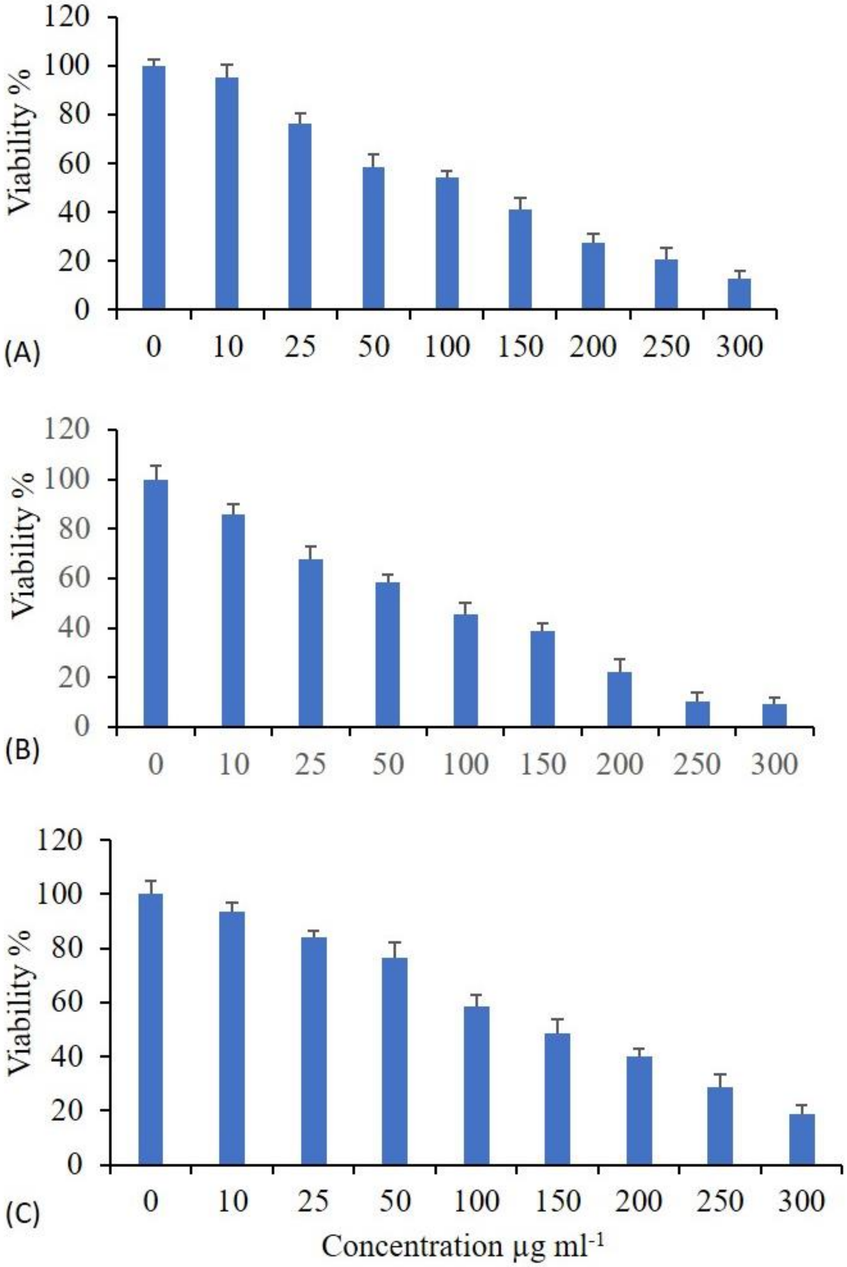

Supplementary Figure 1. Cell viability assay. (A) HepG2 cell lines. (B) HeLa cell lines. (C) McF-7 cell lines.

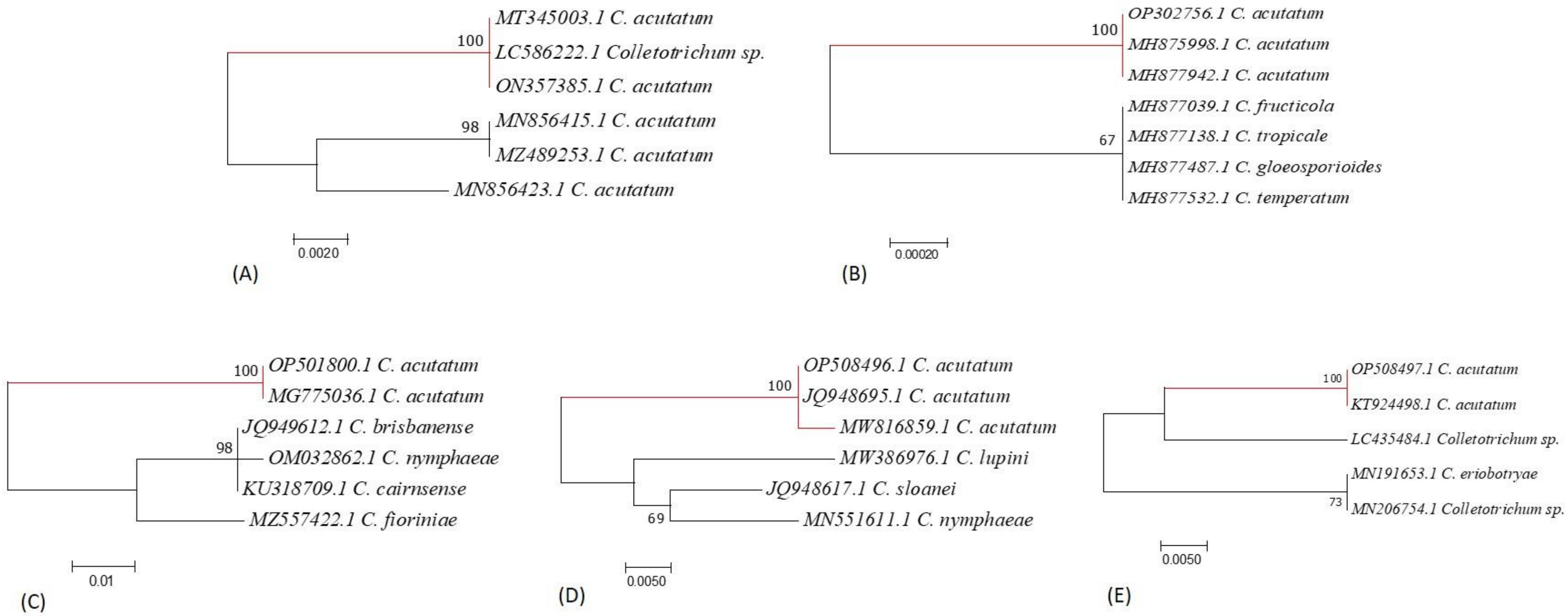

Supplementary Figure 2. Molecular phylogenetic analyses of *C. acutatum* by maximum likelihood method based on the Tamura-Nei model in MEGA7. (A) ITS region. (B) 28S rRNA gene. (C) ACT gene. (D) GAPDH gene. (E) CHS gene.
